# Epidemiology, outcomes and prognosis of infective endocarditis in Northern Morocco

**DOI:** 10.1186/s12879-024-09436-4

**Published:** 2024-07-15

**Authors:** Badre El Boussaadani, Ilias Soussan, Houssam Bendoudouch, Loubna Hara, Amine Ech-chenbouli, Zainab Raissuni

**Affiliations:** Cardiology Department, Road of Rabat, Mohamed VI University Hospital Center Tanger-Tetouan-Al Hoceima, Abdelmalek Essaadi University, Km 17, BP 398 – Gzinaya, Tangier, Morocco

**Keywords:** Infective endocarditis, Valve, Blood culture, Complications, Antibiotic, Surgery

## Abstract

**Introduction:**

Infective endocarditis is a rare but potentially severe disease, associated with significant morbidity and mortality. Our study aims to describe the epidemiology and management aspects of endocarditis in northern Morocco and compare it with international management guidelines.

**Materials and Methods:**

This is a retrospective study involving all patients hospitalized in the cardiology department of the University Hospital of Tangier for infective endocarditis over a period of 4 years and 7 months, from May 2019 to February 2024.

**Results:**

Eighty patients were hospitalized for IE during the study period. The average age of the patients was 46 years, with an even sex ratio. IE concerned native valves in 77% of cases, mechanical prostheses in 19% of cases, and on bio prostheses in 4%. The average diagnostic delay was 25 days. Blood cultures were negative in 59% of cases. The predominant infective microorganism was the bacteria Staphylococcus (65.6%).

Imaging results showed vegetations in 76.3% of cases, predominantly on the mitral valve (39.3%), followed by the aortic valve (21.3%). The main complications included heart failure (51.2%), peripheral arterial embolisms (22.5%) and splenic infarction (17.5%).

Management wise, the most commonly used antibiotic therapy was a combination of ceftriaxone and gentamicin. Clinical and biological improvement was observed in 70% of cases, with a mortality rate of 12.5%. Twelve patients underwent surgery (15%). Urgent surgery was indicated in 66,7% of the operated patients.

**Conclusion:**

Our study highlights the challenges in managing infective endocarditis in northern Morocco. The prognosis of infective endocarditis can be improved through multidisciplinary management within the implementation of an Endocarditis Team.

## Highlights

­ - Infective endocarditis is a rare but severe disease. Guidelines are updated frequently to perfect the assessment and management of this condition.

­ - Complications of IE should be assessed systematically to improve prognosis

­ - Our study proves that the epidemiological data in the guidelines concerning countries where rheumatic heart disease is endemic is similar and compatible with our results.

## Introduction

Infective endocarditis (IE) refers to lesions of cardiac valve endocardium by virulent microorganisms, most often bacterial, and concerns much less commonly non-valvular endocardium, intracardiac prosthesis (IE on prostheses) and devices [1].

It is a rare and serious condition, with significant morbidity and mortality. Its diagnosis is based on a set of clinical and paraclinical arguments. Guidelines have been updated recently by different international committees, such as the 2023 European Society of Cardiology (ESC) guidelines. Diagnosis of IE distinguishes different parameters for native valves, valvular prostheses, and intracardiac devices. Among IE on native valves, the left heart is most frequently affected, and rarely the right heart is affected [2].

The epidemiology of IE is changing, with usual patients with degenerative valve disease being added to those with prosthetic valves and intracardiac devices, and the microbiology is also changing during the past decade. Treatment is based on prolonged antibiotic therapy adapted to the identified responsible organism, and requires thorough assessment of IE complications.

The management of IE must be collaborative, involving the cardiologist, infective disease specialist, cardiovascular surgeon, and sometimes vascular surgeon, intensivist, microbiologist, and possibly other specialists.

The objective of our study is to evaluate the epidemiological, clinical, biological, and prognostic aspects of infective endocarditis in the cardiology setting in Northern Morocco, and to compare our results with the data from the literature and current recommendations.

## Material and methods

This is a retrospective descriptive study including all patients hospitalized in the cardiology department over a period of 4 years and 7 months, from 2019 to 2024. All patients diagnosed with infective endocarditis during this period were included without any exclusion criteria. A total of 80 cases of confirmed infective endocarditis were identified according to the Modified Duke Criteria of the 2023 ESC guidelines for the management of endocarditis.

Patient information was collected from medical records and inscribed using a data collection form. Anonymity and confidentiality of the collected data were strictly maintained. The variables studied in the data collection form include: Epidemiological and socioeconomic data, clinical and paraclinical aspects, (laboratory tests, radiology), complications, follow-up, medical and surgical management and mortality. The data was analysed using "IBM SPSS Statistics 27" statistics software. No artificial intelligence (AI) was utilized in the process.

## Results

### Patient characteristics

The average age of our study group was 46 years [12;78]. The sex ratio was even, with 40 female patients and 40 males.

**Fig. 1:**
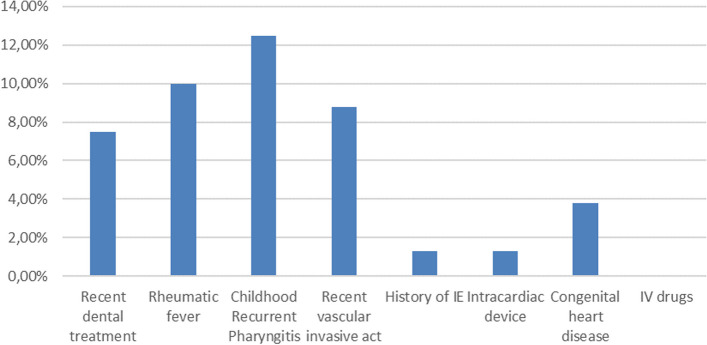
Background risk factors

Background risk factors favouring IE are shown in Fig. 1.

Childhood recurrent pharyngitis was defined by more than 3 episodes of pharyngitis/tonsilitis in one year, before the age of 14. 80% patients with a history of diagnosed rheumatic fever developed carditis before the diagnosis of IE. No patient was diagnosed for degenerative valvular disease before diagnosis of IE. Congenital heart disease cases included 2 cases of bicuspid aortic valve, one case of unoperated persistent ductus arteriosus, and one case of operated single ventricle (tricuspid atresia). were the most common elements. No patient presented with a history of intravenous drug use. Recent vascular act was defined by usage of veinous or arterial central catheter in the past 3 months.

Childhood recurrent pharyngitis and rheumatic fever were the most common risk factors found in our population.

**Fig. 2:**
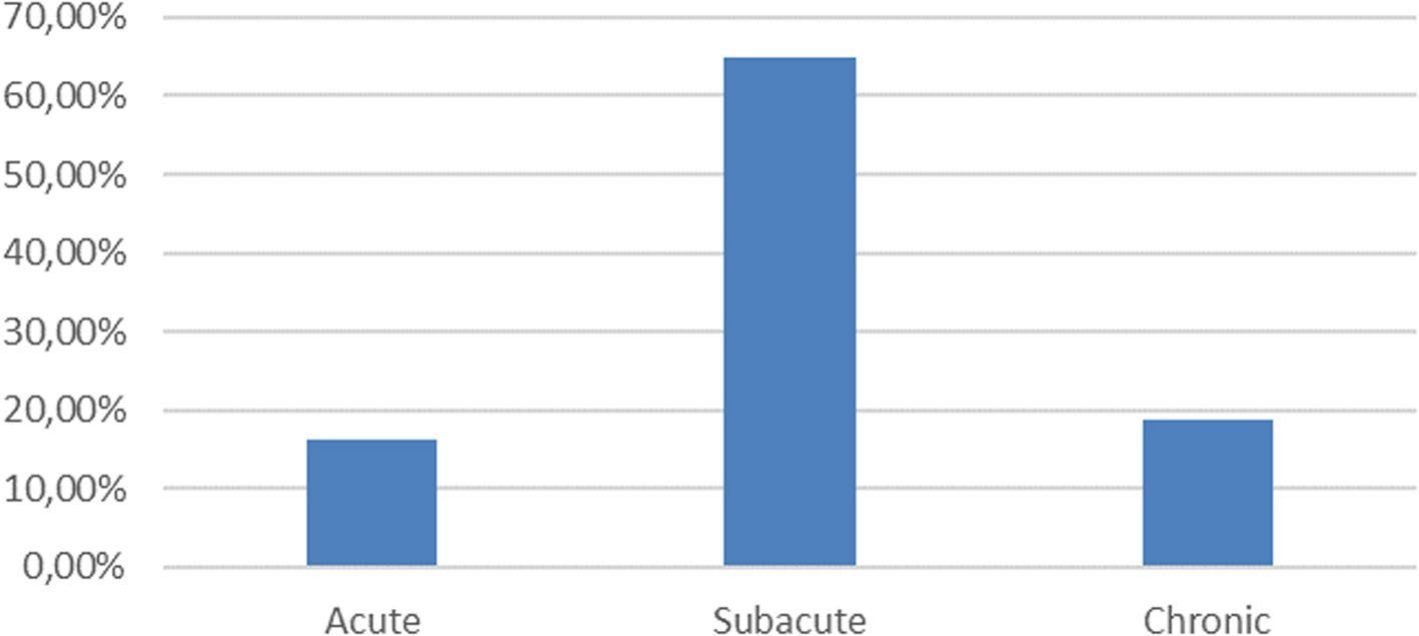
Onset type of IE

The average diagnostic delay is 25 days. IE was subacute in 65% of cases, acute in 16.3% and chronic in 18.8% of patients. (Fig. 2).

### Clinical characteristics

#### Symptoms

The symptoms investigated in our study are: dyspnoea, chest pain, palpitations, joint pain, and malaise, and infective history signs (fever, cough, urinary burning, and digestive symptoms). Poor general condition is defined by the coexistence of at least 2 elements of the following: Fatigue, weight loss and appetite loss. The results are summarized in (Table 1)*.* Almost all patients had fever and poor general condition on admission.

**Table 1:** Symptoms at admission

Symptoms	Percentage
Dyspnoea	65%
Chest pain	8.80%
Palpitations	5%
Joint Paint	8.80%
Poor general condition	87.50%
Fever	90%
Cough	23.80%
Digestive symptoms	8.80%
Urinary burning	1.30%

#### Physical exam elements

The average systolic blood pressure is 112 mm Hg, and the diastolic blood pressure is 65 mm Hg. The average heart rate is 92 beats per minute.

**Fig. 3:**
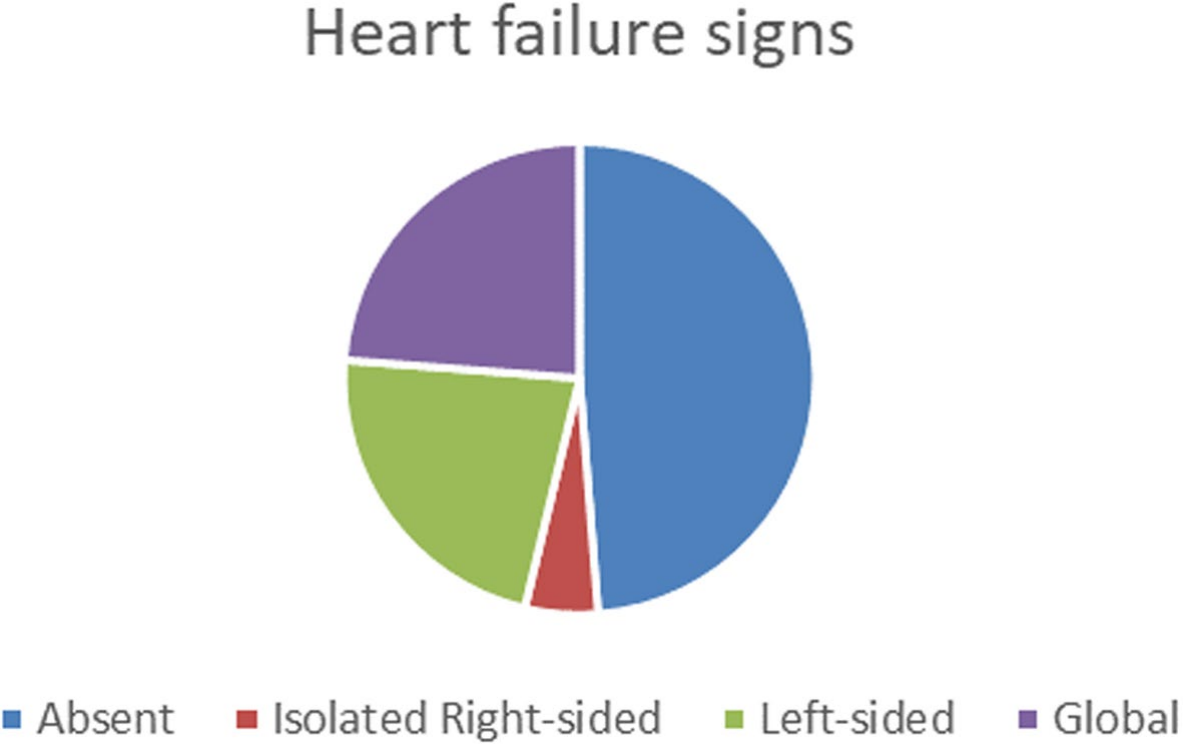
Heart failure signs and profiles

Heart failure congestive signs were present in 51,2% of cases, distributed as following (Fig. 3): left-sided signs (22.5%), global (both left and right signs) 23.8%, and isolated right-sided (5%).

Heart auscultation revealed a regular heart rhythm in 73.8% of patients, with a murmur present in 77.5% of patients (regardless of location). 66,3% of cases presented with bad dental status.

#### Extra cardiac manifestations

Extra cardiac signs and phenomena can be classified as following:

- Vascular phenomena (arterial emboli, peripheral infarcts (kidney, spleen, pulmonary)), assessed by target organ CT imaging.
- Neurological phenomena (intracranial haemorrhage, cerebral mycotic aneurysms), assessed by target organ CT imaging.
- Cutaneous phenomena (Janeway lesions, Osler's nodes, purpura).
- Ocular phenomena (Roth's spots, conjunctival haemorrhage), assessed by fundus examination when patients presented with compatible ocular symptoms.
- Splenomegaly, assessed by physical examination and abdominal CT scan imaging.
- Glomerulonephritis, assessed by dosage of serum complement and eventual kidney biopsy, if the indication is met.

Results are listed in (Table 2).

**Table 2:** Extra cardiac manifestations in our population

Extra cardiac manifestations	Percentage
Vascular phenomena	2.50%
Neurological phenomena	10%
Cutaneous phenomena	Purpura: 6,3% Osler's nodes: 2,3%
Ocular phenomena	1.30%
Splenomegaly	12.50%
Glomerulonephritis	0%

### Diagnosis and types

#### Types of IE

**Fig. 4:**
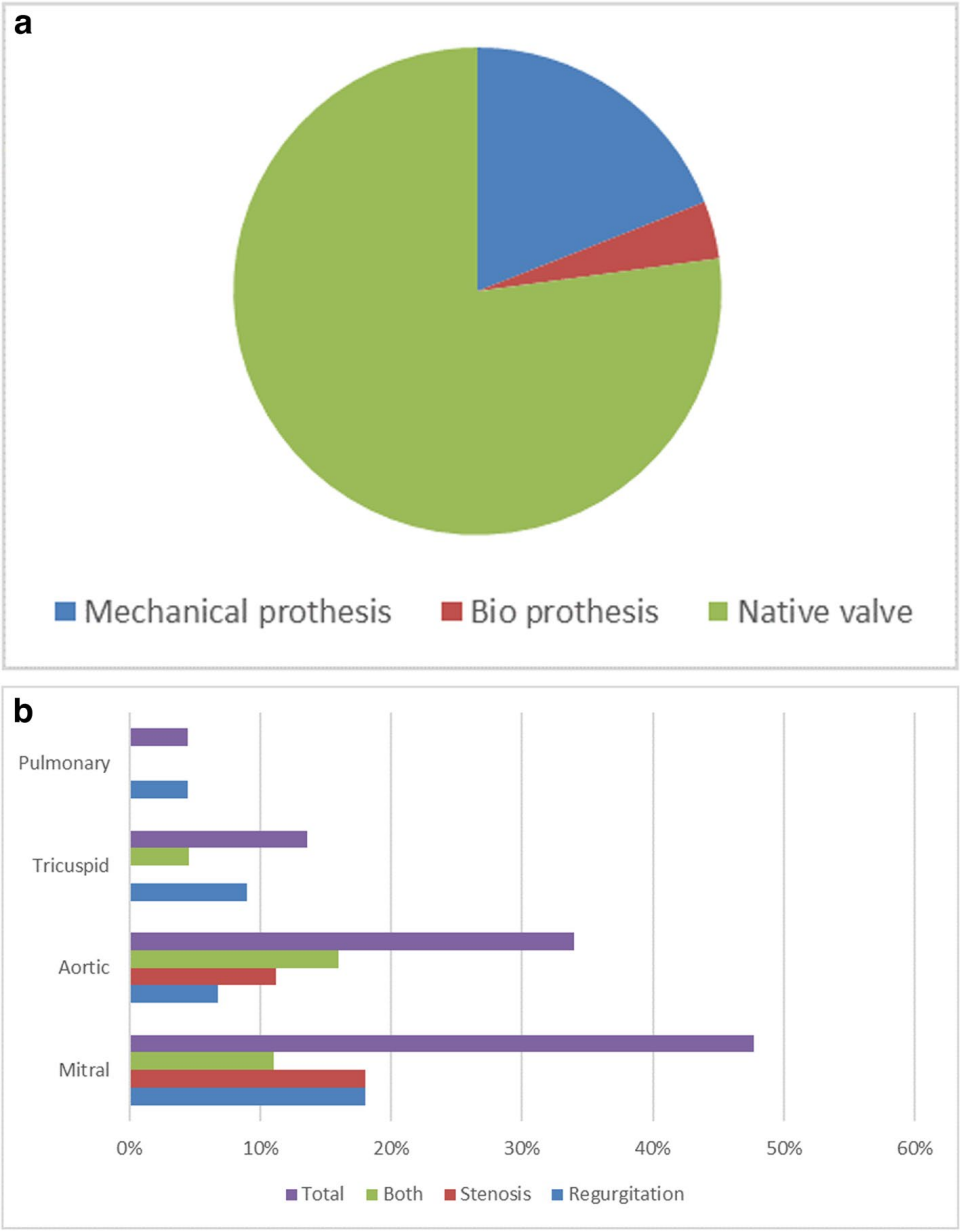
**a** Distribution of IE by native or prosthetic valves. **b** Heart valves types affected in by IE

Seventy seven percent had infective endocarditis on native heart valves, whereas 19% of the cases concerned mechanical valve protheses, and only 4% of bio prothesis IE were found. (Fig. 4a).

Amongst patients with native valves, the mitral valve is the most affected (47,7%), followed by the aortic valve (34%). 8 patients (18%) presented with right-sided native valve IE, with 13,6% presenting with tricuspid valve IE, and two cases of pulmonary valve IE were reported during the study period, with one case being associated with persistent ductus arteriosus. (Fig. 4b).

#### Echocardiogram findings

Transthoracic echocardiography (TTE) was performed on all patients. Transoesophageal echocardiography (TEE) was performed in 61.3% of patients. (38,7% did not benefit from TEE either because of refusal or intolerance of the probe).

Vegetations were found in 76.3% of cases. The average size was 9.85 mm, with a maximum size of 33 mm and a minimum size of 2 mm. They were located on the mitral valve in 39.3% of patients, aortic valve in 21.3% of cases, mitro-aortic in 18% of patients, tricuspid in 9.8% of patients, and pulmonary in 3.3% of patients. They were identified in prosthetic valves in 6.6% of patients.

Valvular and paravalvular abscess was found in 11.3% of cases in our study. Other destructive lesions were identified: 8 patients had chordae tendineae rupture, and 10 patients had valve perforation. We also observed mitral valvular aneurysm in 2.5% of patients.

Prosthesis detachment was diagnosed in 4.9% of cases. Lastly, pericardial effusion was present in 10.1% of patients.

As for other echocardiographic parameters, LV dysfunction is reported in 37.5%, LV dilation in 25%, LA dilation in 42.5%, intra-LA thrombus in 10%, ascending aorta dilation in 5%, pulmonary hypertension (HTAP) in 65%, IVC dilation and decreased compliance in 16.3% of cases.

### Microbiological data

Blood cultures were sampled for all our study patients. Consequently, they were positive in 41.3% of cases. All patients with positive blood cultures had vegetations in their echocardiogram exam, thus 41% of our patients fulfilled two major criteria for IE.

The remaining confirmed cases of IE were considered “definite” with one major criterion and three minor criteria (Modified Duke Criteria 2023).

**Fig. 5:**
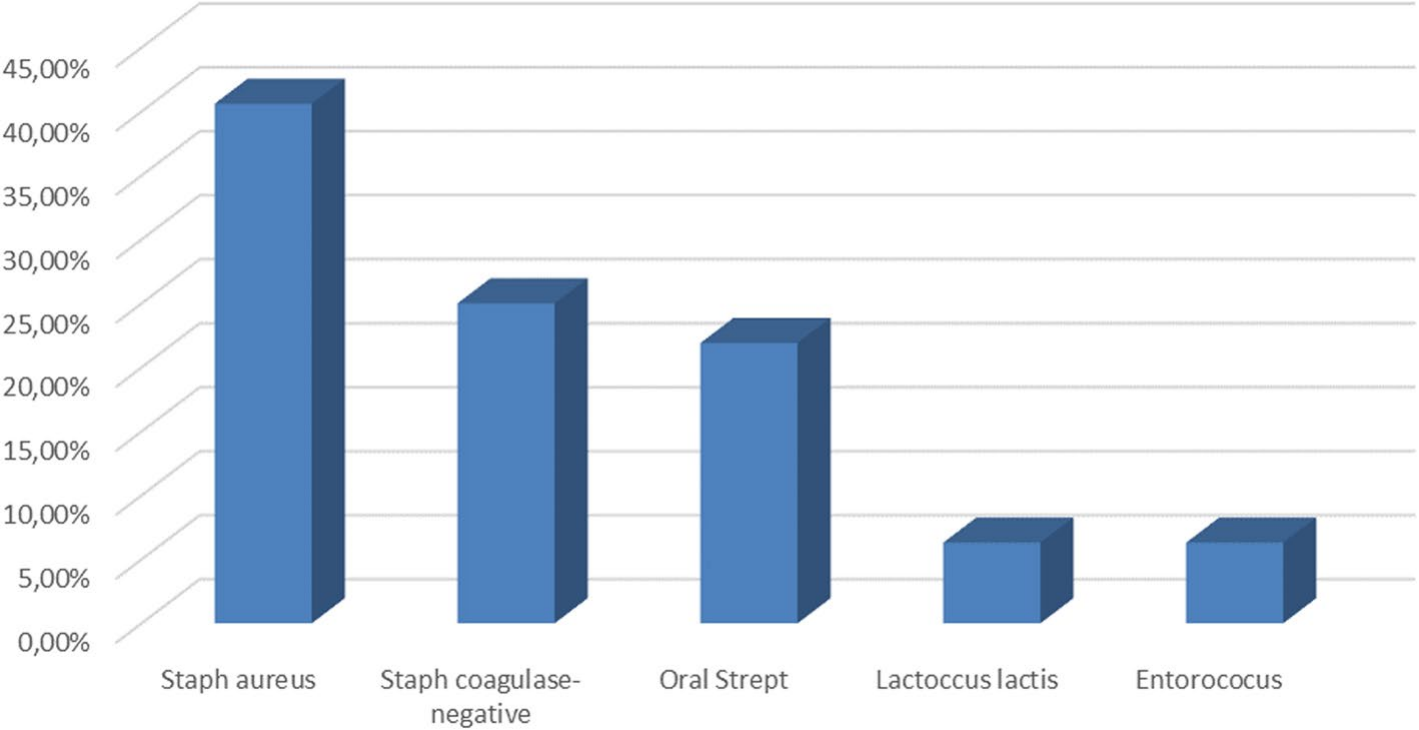
Microbiological blood culture results

The isolated pathogens are as following (Fig. 5): Staphylococcus (65.6%) (Staph. Aureus (40.6%), coagulase-negative Staphylococcus (25%)), Streptococcus (9%), Lactococcus lactis (6.3%) and Enterococcus (6.3%). Isolated Streptococcus species are as following: 3 cases S. gordonii, and 1 case of each of S. oralis, S. mitis, S. sanguinis and S. pneumoniae.

### Therapeutical management

#### Antibiotics

All patients received intravenous antibiotic therapy. Empirical management was indicated initially, then was adjusted according to the antibiogram after blood culture results.

**Fig. 6:**
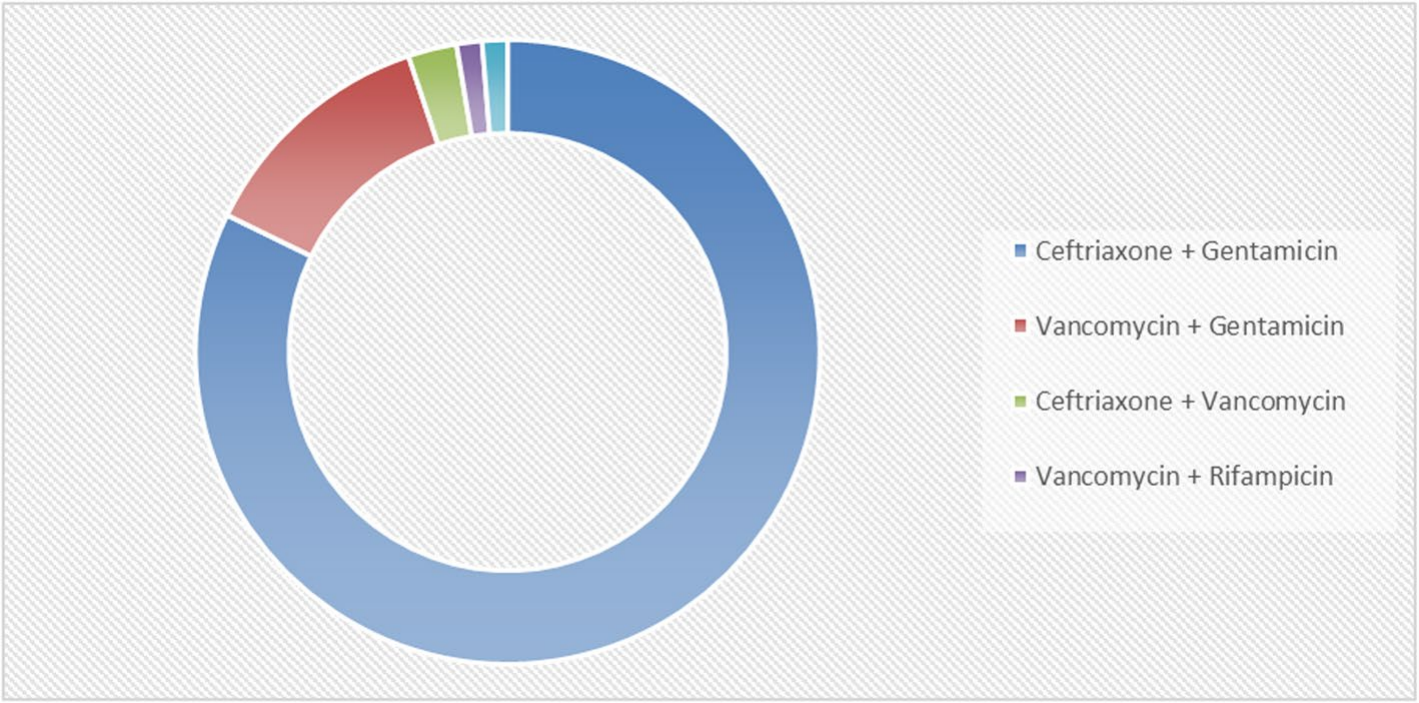
Antibiotherapy in IE

Main initial treatment consisted of a combination of 2 bactericidal antibiotics for an average duration of 4 weeks. The double therapy combinations used in our study are as follows (Fig. 6).

Eighty two percent of patients with native valve IE benefited from empiric protocol using 3rd generation cephalosporin (Ceftriaxone) with Gentamicin. 18% benefited from Vancomycin and Gentamicin. As for prophetic valve IE, 67% benefited from Ceftriaxone + Gentamicin, and 33% benefited from Vancomycin + Gentamicin.

#### Surgical management

A total of 12 patients benefited from surgical management. The degree of urgency of surgical management is defined following the 2023 ESC guidelines: Emergency (8,3%), Urgent (66,7%) and non-urgent (25%).

The characteristics of the *urgent* group is detailed in Table 3.

**Table 3:** Surgical indications and management

Urgent surgery indication:	Percentage	Outcomes (n of patients)
Hemodynamic (Congestive heart failure)	33,3% (4 patients)	1: Improvement 3: Non improvement
Embolic complications	41,7% (5 patients)	2: Improvement 3: Non improvement
Infective (uncontrolled infection)	25% (3 patients)	1: Improvement 2: Non improvement

### Evolution and prognosis

**Fig. 7:**
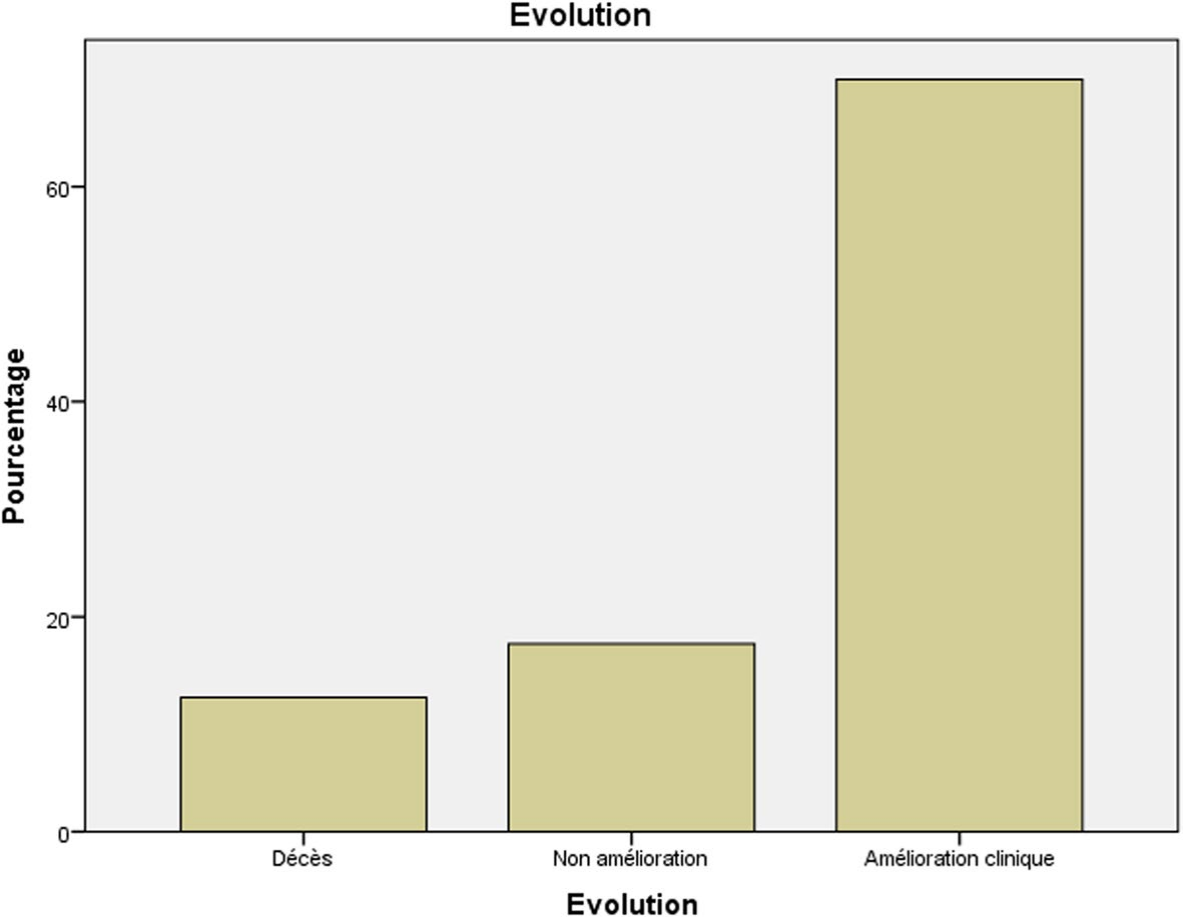
Prognosis of our study population

Clinical and biological improvement was observed in 70% of cases, and no improvement was seen in 17.5% of cases. Death occurred in 12.5% of cases. (Fig. 7).

**Fig. 8:**
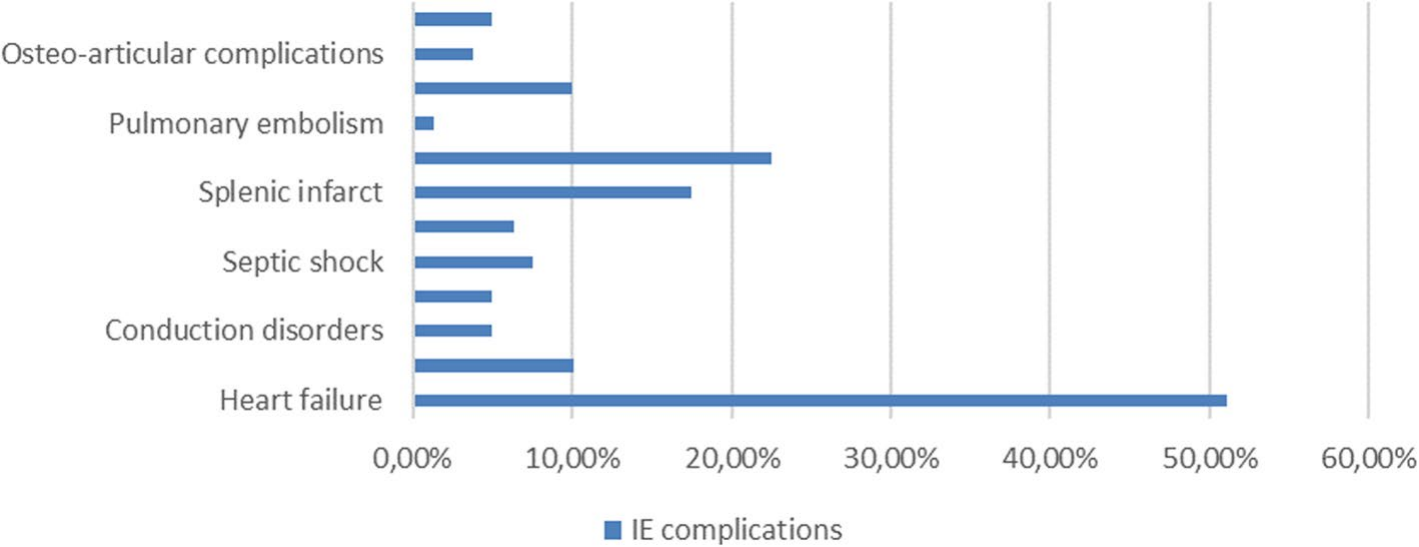
Complications of IE

Complications of infective endocarditis were observed and listed in Fig. 8. Heart failure is the most common complication (51%), followed by peripheral arterial embolization (23%) and splenic infarct (18,5%).

As for patients managed with urgent surgery, outcomes were as follows: 1 patient out 4 improved after surgery for hemodynamic indication, 2 out 3 for embolic indication, and 1 our 3 for infective indication.

## Discussion

### Epidemiology

Our study shows that IE predominantly affects younger individuals with an average age of 46 years. A similar observation is made in Cameroon, as mentioned in the study by Jérôme Boombhi et al. [3] , with an average age of 44 years, and in Algeria, the study by Bennata et al. [4] reports an average age of 40.5. However, the series by Toyoda et al. reports an average age of 62 [5] . In France, as mentioned previously, infective endocarditis is increasingly affecting elderly individuals, with the average age rising from 58 to 62 years between 1991 and 2008.

In our study, infective endocarditis is equally common in men and women. In the series by Toyoda et al., 59% of endocarditis cases were in women [5] . However, a male predominance was noted in the series by Fedeli et al. (62%) [6].

Recent dental care (7.5%) and acute rheumatic fever (10%) are the main risk factors for infective endocarditis according to our study. In the study by Boombhi et al., rheumatic valvular disease represents the main cause of infective endocarditis followed by degenerative valvular heart disease [3] . Similarly, in the study by Bennata et al., rheumatic heart disease account for 70% of cases [4]. Additionally, 33% of patients have a history of rheumatic heart disease according to Blanchard et al. [7].

In contrast, in the Toyoda series conducted in New York, recent valve surgery and the presence of intracardiac material are major risk factors for infective endocarditis [5].

Infective endocarditis on native valves is more common than on prosthetic valves according to our study (77% vs. 23%). This aligns with most series.

### Diagnosis

#### Interrogation and clinical aspects

Interrogation must be thorough to detect risk factors for infectious endocarditis.

Infectious endocarditis can be acute, rapidly progressive, subacute, or even chronic. The average treatment time in our study is 25 days. In a study conducted in Rabat by S. Harrak, the average time required for diagnosis is 77 days [8].

Infectious endocarditis can be diagnosed based on symptoms such as fever, joint pain, general malaise (anorexia, asthenia, weight loss), or cardiac signs such as dyspnoea, chest pain, and palpitations. In our study, fever is present in 90% of cases, while in the series by Duval, 86% of patients are febrile [9] . Boombhi's study shows that all patients have fever [3].

In our series, general malaise is found in 87.5% of patients. Splenomegaly associated with general malaise was present in 11% of cases in the study by Montassier conducted in France [10] . Symptoms found in our patients include dyspnoea in 65% of cases, cough in 23.8% of cases, and chest pain in 8.8% of cases.

*Heart failure* is found in 51.2% of cases, in Fedeli's study HF is found in 13% [6] , in Duval's study HF is found in 33.8% [9] , and represents 26% in Benatta's study [4] in a Tunisian study HF presents 41.5% [10, 11] .

In our study series, cardiac auscultation revealed that 77.5% of patients had a murmur. In 73.8% of patients, the rhythm is regular.

#### Extra cardiac manifestations

Vascular phenomena are found in 2.5% of cases. Neurological signs in 10% of cases. A lone purpura is noted in 6.3% of cases. Purpura was associated with Osler’s nodes in 1.3% of cases. Ocular phenomena represent 1.3% of cases. Splenomegaly in 12.5% of cases. In Montassier's study, Osler's nodes are found in 5% of cases, Janeway lesions in 5% of cases, conjunctival purpura in 5% of cases, Roth's spots in 5% of cases, and neurological manifestations in 5–20% of cases [10].

#### Echocardiography

In addition to blood cultures, echocardiography is the second major criterion for the diagnosis of IE. It plays a crucial role in for diagnosis, prognosis, and treatment monitoring.

Vegetation is present in 76.3% of cases in our study, while in the study by S. Harrak [8] , it is present in 95% of cases. It predominantly occurs at the mitral level in our study, with a percentage of 39.3%, but according to the study by S. Harrak [8] , vegetation predominates at the aortic level with a percentage of 46%, followed by the mitral level with a percentage of 28%.

Myocardial abscess is present in 11.3% of cases in our study, according to S. Harrak [8], it is present in 25% of cases.

As for other destructive lesions 10% of patients had chordal rupture and 12.5% had perforation, while in the study by S. Harrak [8] , 60% of patients had perforation and 43% had chordal rupture. Mycotic aneurysm is present in 2.5% of patients. Prosthesis detachment is reported in 3.8% of cases, in the Harrak study [8] , it was reported in one patient.

Echocardiography assesses the impact of underlying valvopathies by the following data in our study: S. Harrak found that LV dysfunction is reported in 32%, LV dilation in 64%, LA dilation in 70%, intra-LA thrombus in 6%, ascending aorta dilation in 25%, pulmonary hypertension (HTAP) in 88%, IVC dilation and decreased compliance in 36% [8].

### Microbiology

#### Positivity of blood cultures

Haemocultures were positive in 41,3% of cases in our study. However, in industrialized countries, the proportion of negative blood cultures ranges from 5 to 15%, while the proportion of positive blood cultures is approximately 85% [2, 3] . This issue arises in almost all underdeveloped countries.

#### Microbiology

In our work, the predominant pathogen responsible for infectious endocarditis is Staphylococcus, accounting for 65.6% of cases. This is consistent with the series by Fedeli [6] , with a rate of 42%, and that of Nappi [12] , with a staphylococcal rate of 36.3%. However, in the study by Boombhi in Cameroon, streptococcus predominates as the cause of IE (20%), followed by Staphylococcus (10%) [3] . Streptococcus is in the second line in 22% of cases, with Lactococcus lactis in 22% of cases, and finally, Enterococcus in 11% of cases. The blood cultures in our study did not identify other pathogens that are responsible for IE, as mentioned in the 2023 ESC guidelines [1] . No rare IE-related pathogens were identified in our study. These pathogens include bacteria such as Klebsiella spp., Corynebacterium, Campylobacter, Yersinia, Nocardia, Pasteurella, Listeria and even rarer ones such as Serratia marcescens, Aeromonas Salmonicida [13].

### Treatment aspects

#### Antibiotics

Since the majority of blood cultures are negative (59%), antibiotic therapy has mostly remained probabilistic, based on patient history, medical records, clinical signs, whether IE is present on a native valve or a prosthesis (early or late). echocardiographic findings, and sensitivity profile to common pathogens.

Modification of antibiotics were made based on the antibiogram data when blood culture is positive.

The empiric antibiotic protocols used in the data from the work of S. Harrak are mostly Ampicillin or 3rd generation cephalosporins + gentamicin for native valve IE, and mostly Vancomycin + gentamicin for prosthetic valve IE.

#### Surgery

Fifteen percent of patients underwent early surgery. Among these patients, 41.7% of indications were embolic, 33% were for hemodynamic indications, and 25% were for infectious indications. In the series by S. Harrak, 7% of patients underwent early surgery, and the main indication was mostly hemodynamic [8].

### Prognosis

#### Mortality

According to studies, in-hospital mortality is approximately 15 to 25%, 6-month mortality is 30%, and 5-year mortality is around 40%. These data hardly change over time. This is probably due to the epidemiological developments of IE, with more frequent nosocomial-origin infections affecting older patients with multiple comorbidities [2, 3] . The mortality rate in our series is 12.5%.

#### Prognostic factors

In our study, the prognostic factors found in patients who did not improve or died are shown in Table 4.

**Table 4:** Factors of poor prognosis in IE

Patient characteristics:	Presence of IE complications:	Echocardiographic data:
- Age between 30 and 50 years	- Heart failure (60%)	- Vegetation size > 10 mm (46%)
	-Peripheral arterial embolism (29.2%)	- Valvular prosthesis (21%)
- Male gender	- Septic shock (25%)	Altered left ventricular ejection fraction (54%)
	- Splenic infarction (25%)	Pulmonary arterial hypertension (54%)
	-Cardiogenic shock 16.70%	

### Summary chart

Following the results of our study, we summarize the characteristics of our population in Table 5.

**Table 5:** Patient profile summary chart

Characteristics	Results
Young patients	Mean age: 46 years old
No sex predominance	Sex ratio 1:1
Rheumatic fever	10%
Recurrent pharyngitis	12,5%
Diagnosis delay	Mean: 25 days
Initial infection non identified	72,5%
Fever	90%
Vegetations	76,3%
Types of IE	MS 10% MR 10% MD 6,3% AS 6,3%, AR 3,8%, AD 8,8%
Negative blood cultures	59%
Heart Failure	51,2%
Splenomegaly	12,5%
Neurological complications	10%
Clinical improvement	70%
Death	12,5%
Urgent surgery	15%

## Limits of the study

- Retrospective data collection.

## Conclusion

IE is a serious condition with significant morbidity and mortality. Unfortunately, its frequency has not decreased in recent years. In our study, the epidemiological profile shows minimal change regarding the predominance of Staphylococcus as the responsible germ for IE, reflecting the minimal decrease in IE on rheumatic valves.

Its complications are numerous and fatal, and sometimes early valve surgery is necessary in cases of indication to improve prognosis. Disease prevention is a crucial step that requires a more serious application of prevention rules as well as better diagnostic and therapeutic management of the patient at the first symptoms of endocarditis to reduce the frequency and severity of the disease.

In our context, there are several difficulties to face in order to improve the prognosis of patients with IE, such as diagnostic delay, difficulty in identifying the portal of entry, frequency of negative blood cultures, and incomplete extension assessment. Its diagnosis remains challenging and represents a challenge for all practitioners. Its management requires multidisciplinary collaboration within an Endocarditis Team.

## Data Availability

The datasets used and/or analysed during the current study are available on reasonable request from the corresponding author: Badre El Boussaadani.
